# Saudi plastic surgeons’ venous thromboembolism prophylaxis practices in aesthetic surgery: A national study

**DOI:** 10.1016/j.jpra.2022.06.004

**Published:** 2022-06-26

**Authors:** Khalid Arab, Salman S. Qasim, Hatan Mortada, Abdullah A. Al Qurashi, Alaa Mohammed AlSahli, Jumanah T. Qedair, Hisham Alghamdi, Abdullah Kattan

**Affiliations:** a Division of Plastic Surgery, Department of Surgery, College of Medicine, King Saud University, Riyadh, Saudi Arabia; b College of Medicine, King Saud bin Abdulaziz University for Health Sciences, Ar Rimayah, Riyadh 14611, Saudi Arabia; c Department of Plastic Surgery & Burn unit, King Saud Medical City, Riyadh, Saudi Arabia; d College of Medicine, King Saud bin Abdulaziz University for Health Sciences, Jeddah, Saudi Arabia; e King Abdullah International Medical Research Center, Jeddah, Saudi Arabia

**Keywords:** Prevention, Prophylaxis, Thromboembolism, Aesthetic

## Introduction

The diagnosis of deep vein thrombosis (DVT) or pulmonary embolism (PE) is referred to as venous thromboembolism (VTE).^1^ VTE is considered one of the most serious and feared complications following aesthetic plastic surgery.^2^ It was previously recognized as a public health crisis by The United States Surgeon General and is the leading cause of mortality following cosmetic surgery.^3^

Aesthetic plastic surgeons' VTE prophylactic practices remain unknown, and data regarding the implementation of VTE preventive measures are lacking.^4^^,^^5^ Hence, this study aims to detail the VTE preventive practices amongst Saudi aesthetic surgeons, and to decide whether the routinely implemented VTE preventive practices of Saudi aesthetic surgeons meet the internationally recognized standards or not.

## Methods and materials

The questionnaire was revised by three plastic surgery consultants to ensure the quality and objectivity of the items. In addition to the questions targeting demographic data, questions aimed at assessing prophylaxis practices included the type and frequency of VTE prophylaxis used, post-operative VTE screening and management, the determinants of choosing VTE prophylaxis modalities, and utilization of risk stratification guidelines.

The survey was distributed online to aesthetic surgery consultants practicing in Saudi Arabia. Data was from the 15th of October until the 13th of November. The inclusion criterion of our study was being a plastic surgery consultant and practicing in Saudi Arabia during the study period. Responses from non-plastic surgery consultants, or plastic surgery consultants who were not practicing in Saudi Arabia were excluded.

Descriptive statistics were used to describe the overall group of respondents, and they have been presented using frequency and percentages (%). A multiple response analysis was performed to analyse participants’ responses.

## Results

The valid responses of 48 plastic surgeons were analysed. Approximately one-third of respondents were experienced surgeons (31.3%), and 50.0% of them were performing >50 aesthetic operations per year. The demographic and occupational characteristics of participants are listed in Table 1.

**Table 1:** Demographic and occupational characteristics of the respondents (*n* = 48).

Variable	Category	Frequency (*N* = 48)	Percentage (%)
Age	30 to <40	19	39.6
	40 to <50	15	31.3
	50 to <60	8	16.7
	≥60	6	12.5
Gender	Male	41	85.4
	Female	7	14.6
Residency Training program	Saudi	22	45.8
	French	5	10.4
	American	17	35.4
	Other	4	8.3
Years in practice	0–5	15	31.3
	5–10	13	27.1
	10–15	5	10.4
	>15	15	31.3
	0–10	1	2.1
	11–25	9	18.8
Number of aesthetic operations per year	26–50	14	29.2
	>50	24	50.0
Number of aesthetic operations per year Region of practice	Central	26	54.2
	Western	14	29.2
	Eastern	7	14.6
	Southern	1	2.1
	Private practice (solo or group)	11	22.9
	Academic	16	33.3
Primary practice setting	Non-academic hospital employed	19	39.6
	Other (corporate or multispecialty group)	2	4.2

DVT prophylaxis was consistently used by 50% of the respondents all the time, whereas 37.6% of the respondents have adopted prophylactic approaches for 50–75% of the time. The most frequently used modalities for DVT included stockings (*n* = 25, 23.4%), a combination of low-molecular-weight heparin (LMWH) and SCD (*n* = 21, 19.6%) and SCDs alone (*n* = 11, 10.3%).

Only 13 surgeons (27.1%) routinely screened for VTE prior to discharge or within the first month of the postoperative period. Physical examination was the most frequently used approach for screening of a DVT or PE (47.2%), while ultrasonography or CT/MR angiogram were almost equally utilized by the participants (23.6% and 19.4%, respectively). Further details of the screening and management of asymptomatic VTE are presented in Table 2.

**Table 2:** Participants’ responses regarding their practices of screening and Management of asymptomatic Venous Thromboembolism (VTE) (*n* = 48).

Variable	Category	Frequency (*N* = 48)	Percentage (%)
Do you routinely screen for PE*/DVT⁎⁎ on patients prior to discharge or in acute postoperative period (within 30 days of surgery)?	No	35	72.9
	Yes	13	27.1
Modality used when screening for DVT or PE routinely performed ⁎⁎⁎	Physical exam	34	47.2
	Ultrasound	17	23.6
	CT or MR angiogram	14	19.4
	D dimer	7	9.7
Management of asymptomatic DVT or PE ^¥^	Observation only	4	7.3
	Serial imaging	0	0
	Anticoagulation	20	36.4
	IVC filter placement	0	0
	Referral to pcc, vascular surgeon, haematologist or other specialists	31	56.4
Do you routinely use the same chemoprophylactic modality for patients who require treatment?	No	13	27.1
	Yes	35	72.9

⁎ PE= Pulmonary Embolism.

⁎⁎ DVT= Deep Venous Thrombosis.

⁎⁎⁎ The total number of responses was for the multiple response item was 72 responses; ¥ The total number of responses was for the multiple response item was 55 responses.

Participants’ responses showed that weight bearing restriction was the most influential factor on the of DVT prophylaxis selection (*n* = 39, 81.3%), while the effects of undergoing major surgeries and literature support were similarly expressed by the respondents (*n* = 35, 72.9%). On the other hand, the duration of DVT prophylaxis modalities primarily relied on weight bearing restrictions (*n* = 39, 81.3%) followed by literature support (*n* = 36, 75.0%) and undergoing major surgeries (*n* = 32, 66.7%).

The Caprini 2005 risk assessment score was the most used official risk stratification guidelines (50.0%) for patient assessment followed by the institutional guidelines (22.7%). Additionally, 87.5% of respondents indicated that VTE prophylaxis in the institution/hospital was guided by specific guidelines, of which Caprini 2005 risk assessment score (57.1%) and institutional guidelines (21.4%) were frequently employed.

Testing for coagulation was routinely carried out by more than half of the respondents (62.5%). Focusing on those who responded positively, the primary indication for routine testing included having a personal history of unexplained DVT or PE (53.5%) followed by having a family history of a clotting/bleeding disorder (33.3%).

## Discussion

In terms of DVT prophylaxis, 50% of our cohort reported using at least one modality for the prevention of DVT, with stockings being the most common followed by a combination of low molecular-weight heparin (LMWH) and sequential compression device (SCD) then SCDs alone. By comparison, the aesthetic surgeons in Aimé VL et al.'s study commonly utilized chemoprophylaxis (48.7%) followed by mechanical prophylaxis (35.8%).^5^ The need for a specific type of DVT prophylaxis is mainly determined based on different risk stratification guidelines.^5^ Thus, the difference in the prophylaxis utilized could be due to the different risk stratification guidelines employed in Aimé VL et al.'s cohort compared to ours. Most of the respondents in the present study (91.7%) utilized a patient risk stratification assessment tool, with the Caprini 2005 Risk Assessment Score being the most employed (50%), followed by the institutional guidelines (22.7%). In comparison to Aimé VL et al.'s cohort, 93.6% of their participants utilized a risk stratification assessment tool.^4^ The commonly employed risk stratification assessment tool was Caprini 2005 (74.2%) followed by ASPS VTE prophylaxis task force (36.9%).^6^ A minority of plastic surgeons in the present study (6.85%) depended on their personal experience or preference for determining the most suitable DVT prophylaxis modality while considering patients’ demographic characteristics and history, which is a much lower figure compared to that of the aforementioned study (27.5%).^6^ A possible explanation for this considerable difference is that most of The Aesthetic Society members surveyed worked primarily in private practice, in which institutional risk stratification guidelines may not be routinely employed, compared to our cohort.

Routine testing for coagulopathies before surgery remains a common practice by many surgeons. This standard, unselected coagulation testing is not supported by current data.^7^ More than half of the respondents in the present study routinely tested for coagulopathies prior to surgery. The primary indication for routine testing included having a personal history of unexplained DVT or PE followed by having a family history of a clotting/bleeding disorder. A systematic review conducted by the British Committee for Standards in hematology on the guidelines of blood testing supported this practice and recommended that patients with no bleeding history should not be routinely screened for coagulopathies before surgery.^8^

Many plastic surgeons remain hesitant to use DVT chemoprophylaxis prior to surgery, despite it being one of the most serious and feared complications following aesthetic plastic surgery.^2^^,^^9^ In the literature, the use of chemoprophylaxis amongst high-risk patients is controversial because of the danger of postoperative bleeding and haematoma formation.^9^ In the present study, the most common barrier to the implementation of a chemoprophylactic regimen was the heightened risk of postoperative bleeding or haematoma, followed by the self-perceptions that there is no evidence regarding the usefulness of prophylaxis in aesthetic surgery patients.

The are several limitations to the present study. The first is the nature of cross-sectional surveying, which involves internal bias and recall bias; thus, the findings of the study should be considered cautiously. The second limitation is the inability to calculate a power-analysis, as the actual number of Saudi aesthetic surgeons was unknown to the authors; however, the aim of this study was to provide a beneficial glimpse of the currently employed VTE preventive practices amongst Saudi aesthetic surgeons.

Most of the identified VTE prophylactic measures and techniques of Saudi aesthetic surgeons align with the literature and plastic surgery speciality international association recommendations. However, we identified few practices disparities amongst our cohort compared with international cohorts. More awareness of the internationally implemented VTE preventive practices to Saudi aesthetic surgeons is highly recommended.

## Conflict of interest statement

All authors disclose any financial and personal relationships with other people or organizations that could inappropriately influence (bias) this work.

## Informed consent

Before participation in the research, informed consent was gained from all participants.

## Funding

None.

## Ethical approval

The study was conducted under ethical approval from 10.13039/501100002383King Saud University Institutional Review Board (IRB) (#No. E-21–6362).

## Declaration of Competing Interest

None declared.
